# Supplementary material for: The Sh3Pxd2bnee−/− mouse reveals developmental features of Frank-ter Haar syndrome
Source: Development. 2026 Feb 2;153(2):dev204631. doi: 10.1242/dev.204631 (PMC12912270; doi:10.1242/dev.204631)
Supplement: Supplementary information [file develop-153-204631-s1.pdf]

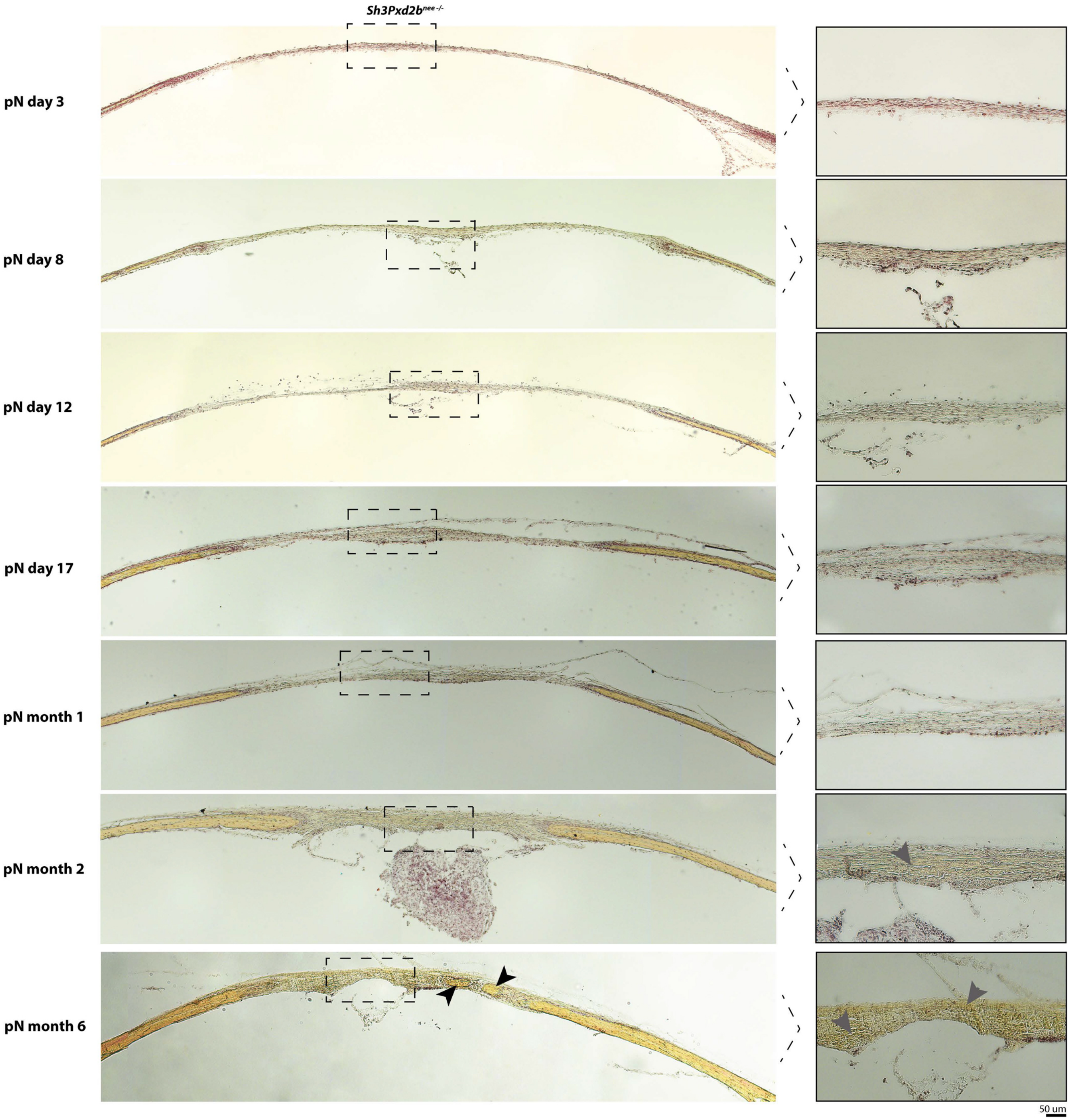

Fig. S1. Sh3Pxd2b$^{nee -/-}$ mice are characterized by disrupted sagittal suture patterning in vivo (A) Movat's Pentachrome staining of the sagittal suture of *Sh3Pxd2b$^{nee -/-}$* mice encompassing both osteogenic fronts. Ectopic bone formation is observed in the Sag suture of mutant mice in pN month 6 (black arrowheads). The framed panels represent magnification of a representative area of the suture mesenchyme, demonstrating increasing ossification starting at two months postnatally (grey arrowheads). Images represent tiled composites of multiple adjacent fields. See also Fig. 1K.

Development • Supplementary information

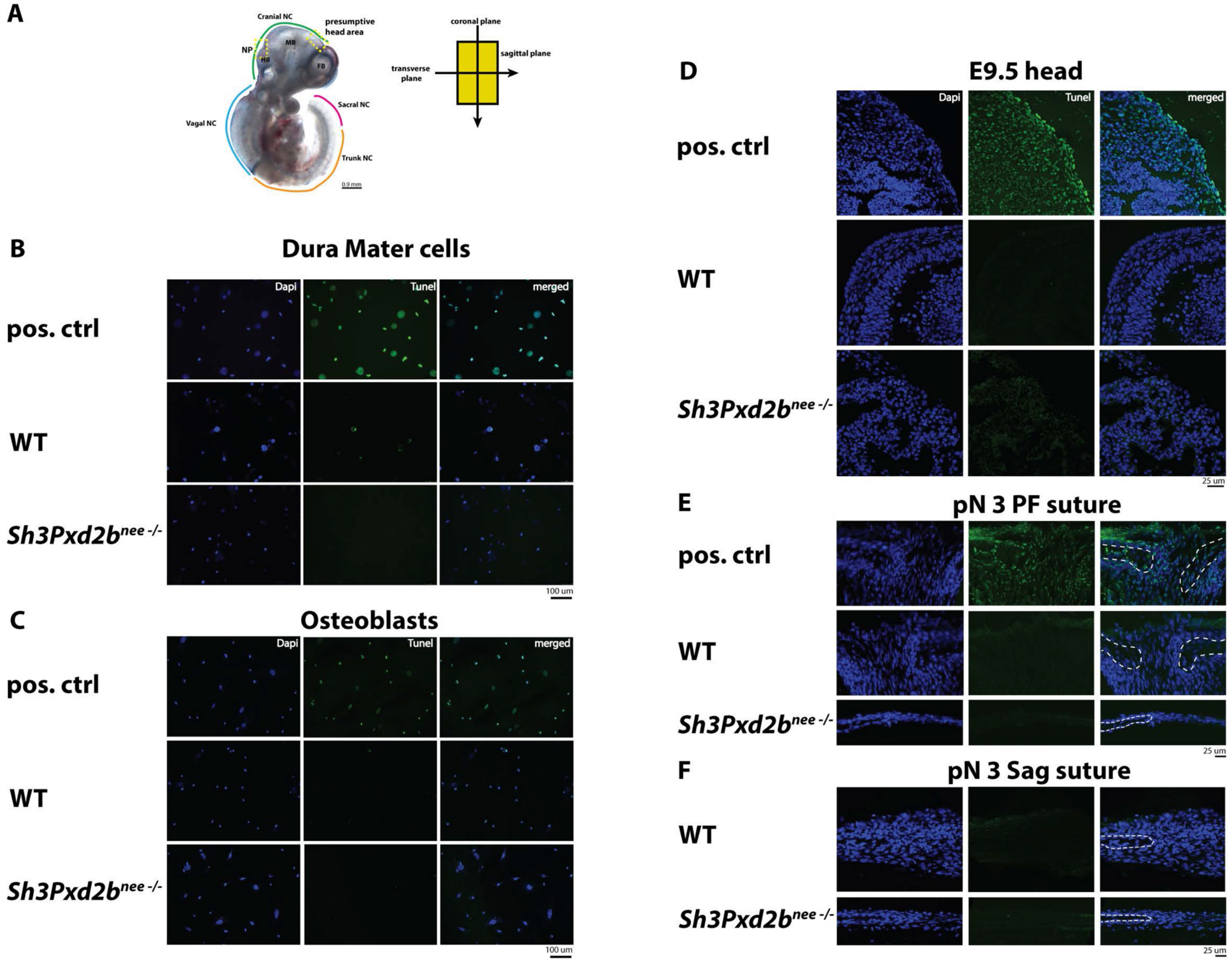

Fig. S2.

(A) E9.5 WT embryo with anatomical landmarks. Yellow dashed rectangles mark the areas selected for imaging of the presumptive head area and the neural plate. A schematic is included to illustrate the orientation of the sagittal, coronal, and transverse planes relative to the embryo for reference. See also Figures 3C, 4D, 5A-D, 7E-F. All referenced images were acquired in the sagittal plane. FB: Forebrain, MB: Midbrain, HB: Hindbrain, NC: Neural Crest. **(B-F) Similar apoptotic activity in vitro and in vivo in WT and Sh3Pxd2b$^{nee\ -/-}$ mice.** Assessment of apoptotic activity using Tunel assay in dura mater cells (B) and osteoblasts (C) from WT and *Sh3Pxd2b$^{nee\ -/-}$* mice *in vitro* and presumptive head area of E9.5 embryos (D) and p3 PF (E) and Sag sutures (F) of WT and *Sh3Pxd2b$^{nee\ -/-}$* mice. Dashed lines mark bone fronts. Positive controls in top panels. No difference of apoptotic activity is observed in any samples at any time point.

Development • Supplementary information

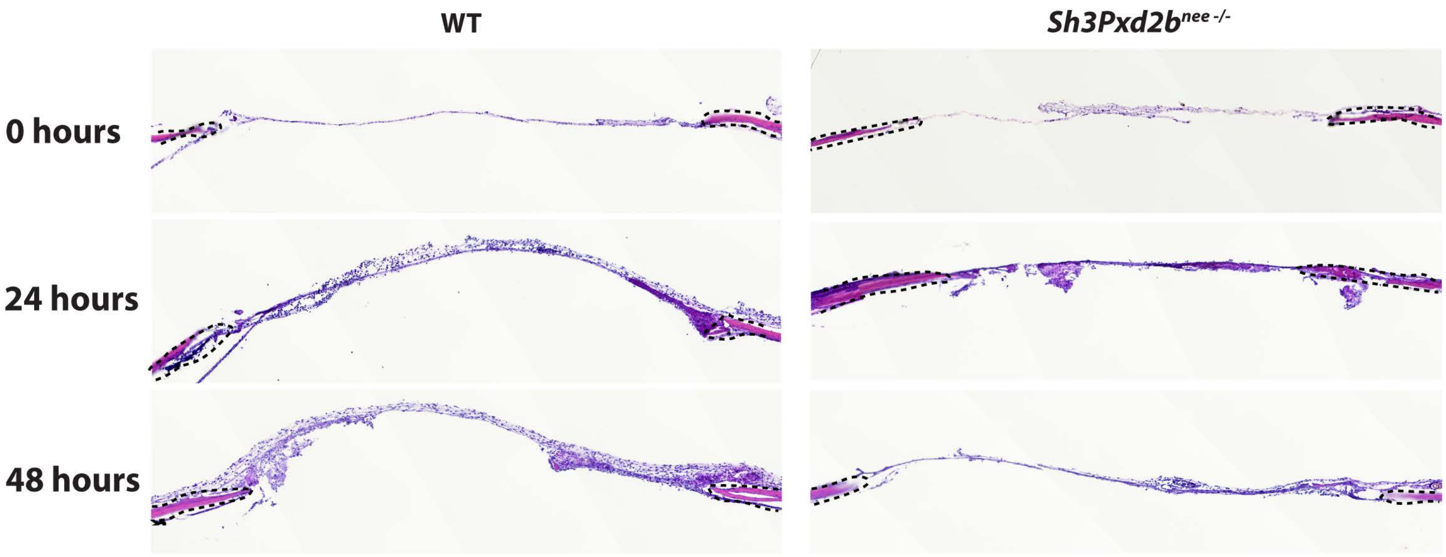

Fig. S3. Calvarial defects in mouse parietal bones. Hematoxalyn&Eosin stained 10 µm sections of calvarial defect area in WT and Sh3Pxd2b$^{nee -/-}$ mice at time of surgery and 24 and 48 hours postoperatively showing the thin layer of the dura mater intact. Bone fronts are marked by dashed lines. At 24 and 48 hours, cells have migrated into the defect area in WT mice, whereas in mutant mice, no migration of cells can be observed. See Figure 4M for magnified sections of representative areas in the defect. Images represent automatically tiled composites of multiple adjacent fields acquired with a digital slide scanner.

Table S1. Table listing KEGG pathway enrichment analysis of genes downregulated in *Sh3Pxd2b*$^{nee\ -/-}$ mouse skulls

Term	Adjusted P value	Odds Ratio	Combined Score
Ribosome biogenesis in eukaryotes	7.41E-49	119.0379	13645.3
Ribosome	5.07E-41	67.3896	6462.34
Gastric acid secretion	0.607718	5.620181	16.51034
cGMP-PKG signaling pathway	0.607718	3.61087	10.45911
Insulin secretion	0.607718	4.814383	12.87033
Proximal tubule bicarbonate reclamation	0.607718	9.561809	21.59923
Fatty acid elongation	0.607718	7.168831	14.33528
Biosynthesis of unsaturated fatty acids	0.607718	6.474096	12.35575
Vascular smooth muscle contraction	0.607718	2.922508	5.445387
Nicotinate and nicotinamide metabolism	0.607718	5.733045	10.32157
Adrenergic signaling in cardiomyocytes	0.607718	2.761252	4.905207
Aldosterone-regulated sodium reabsorption	0.607718	5.422604	9.495593
Carbohydrate digestion and absorption	0.607718	4.775854	7.830035
Mineral absorption	0.607718	4.664553	7.551493
Cocaine addiction	0.607718	4.26671	6.576964
Arginine and proline metabolism	0.607718	4.092146	6.160346
Endocrine and other factor-regulated calcium reabsorption	0.607718	3.712308	5.278844
Graft-versus-host disease	0.607718	3.180536	4.10832
Amphetamine addiction	0.607718	2.99005	3.709113
Bile secretion	0.607718	2.821027	3.364635
Thyroid hormone synthesis	0.607718	2.781706	3.285871
Synaptic vesicle cycle	0.607718	2.634769	2.996335
Cardiac muscle contraction	0.607718	2.60042	2.929772
Salivary secretion	0.607718	2.60042	2.929772
Protein digestion and absorption	0.65472	2.248439	2.273925
Prostate cancer	0.658985	2.083754	1.984794
Aldosterone synthesis and secretion	0.658985	1.980098	1.809155
Pancreatic secretion	0.658985	1.922688	1.7141
Thyroid hormone signaling pathway	0.662405	1.753145	1.443153
Natural killer cell mediated cytotoxicity	0.662405	1.707934	1.373486
Platelet activation	0.662405	1.610948	1.227928
Dopaminergic synapse	0.662405	1.489974	1.054192
Purine metabolism	0.662405	1.478863	1.03869
Apelin signaling pathway	0.662405	1.457126	1.008593
Parkinson disease	0.66264	1.395564	0.925033
Oxytocin signaling pathway	0.674066	1.303691	0.805122
Tight junction	0.691475	1.200803	0.678061
Calcium signaling pathway	0.712159	1.059102	0.516626
Alcoholism	0.712159	1.005102	0.459569
Focal adhesion	0.712159	1.005102	0.459569
cAMP signaling pathway	0.712509	0.94709	0.401212
Regulation of actin cytoskeleton	0.712509	0.920501	0.375523
Neuroactive ligand-receptor interaction	0.866412	0.569179	0.107494
Pathways in cancer	0.955236	0.366322	0.025009
Olfactory transduction	0.997108	0.16747	0.000485

Table S2. Tables listing concentrations of total small RNA, microRNA (~10-40 nucleotides (nt)), 5S RNA (~92-93 nt) and 5.8S RNA (~134-135 nt) and their ratios relative to total small RNA concentration. Data were obtained using the Agilent Bioanalyzer Small RNA assay in RNA extracted from whole mount skulls of postnatal day 15 WT and *Sh3Pxd2b*$^{nee\ -/-}$ mice. Values represent individual biological replicates. Mean and standard deviation (SD) are reported for each sample.

Small RNA concentration (pg/µl)		
	WT	*Sh3Pxd2B*$^{nee-/-}$
**Sample 1**	2887.1	1250.3
**Sample 2**	7312.9	243.9
**Sample 3**	1488.5	778.6
**Sample 4**	1695.1	N/A
**Sample 5**	3081.9	N/A
**Mean (± SD)**	3293.1 (±2106.1)	757.6 (±411.1)

microRNA concentration (pg/µl)		
	WT	*Sh3Pxd2B*$^{nee-/-}$
**Sample 1**	302.8	70.4
**Sample 2**	2203.2	6.9
**Sample 3**	202.6	58.8
**Sample 4**	284.2	N/A
**Sample 5**	602.3	N/A
**Mean (± SD)**	719.0 (±754.4)	45.4 (±27.6)

microRNA (%)		
	WT	*Sh3Pxd2B*$^{nee-/-}$
**Sample 1**	10	6
**Sample 2**	30	3
**Sample 3**	14	8
**Sample 4**	17	N/A
**Sample 5**	20	N/A
**Mean (± SD)**	18.2 (±6.8)	5.7 (±2.1)

5S RNA concentration (pg/µl)		
	WT	*Sh3Pxd2B*$^{nee-/-}$
**Sample 1**	664.6	269.4
**Sample 2**	1027.9	60.2
**Sample 3**	231.9	162.0
**Sample 4**	251.3	N/A
**Sample 5**	451.2	N/A
**Mean (± SD)**	525.4 (±296.2)	163.9 (±85.4)

5S RNA ratio (%)		
	WT	*Sh3Pxd2B*$^{nee-/-}$
**Sample 1**	23	22
**Sample 2**	14	25
**Sample 3**	16	21
**Sample 4**	15	N/A
**Sample 5**	15	N/A
**Mean (± SD)**	16.4 (±3.3)	22.3 (±1.7)

5.8S RNA concentration (pg/µl)		
	WT	*Sh3Pxd2B*$^{nee-/-}$
**Sample 1**	837.90	282.0
**Sample 2**	785.6	85.7
**Sample 3**	179.8	172.6
**Sample 4**	194.1	N/A
**Sample 5**	356.3	N/A
**Mean (± SD)**	470.7 (±285.7)	180.1 (±80.3)

5.8S RNA ratio (%)		
	WT	*Sh3Pxd2B*$^{nee-/-}$
**Sample 1**	29	23
**Sample 2**	11	35
**Sample 3**	12	22
**Sample 4**	11	N/A
**Sample 5**	12	N/A
**Mean (± SD)**	15.0 (±7.0)	26.6 (±6.0)

Development • Supplementary information

Table S3. Table depicting the top 50 most differentially expressed upregulated genes between mutant mouse genes, showing genes involved in metabolic and biochemical cellular processes.

		logFC
1	Bpifb4	10.27792
2	Sult1c1	10.10045
3	Cyp1a2	10.03957
4	Pitx1	9.947594
5	Umodl1	9.878106
6	Chil6	9.698676
7	Bpifb9a	9.616918
8	Cyp2a5	9.599082
9	Bpifb5	9.582282
10	Bpifb9b	9.573317
11	BC051076	9.489758
12	Slc5a8	9.029996
13	Gm281	9.022635
14	Pigr	8.962907
15	Obp2b	8.960164
16	Ugt2a2	8.909482
17	Bpifb6	8.901177
18	Ugt2a1	8.845745
19	Cyp2g1	8.824715
20	Wfdc18	8.764159
21	Chil4	8.745826
22	Muc16	8.678697
23	Gpx6	8.618938
24	Obp2a	8.592524
25	Sult1b1	8.562006

		logFC
26	Arhgap40	8.524266
27	Sec14l3	8.513458
28	5430401F13Rik	8.461607
29	BC048679	8.448484
30	Cyp2a4	8.434836
31	Bpifa1	8.407993
32	Lcn11	8.37581
33	Sftpd	8.235703
34	Psapl1	8.229701
35	Bpifa6	8.190732
36	Ascl3	8.151142
37	Mslnl	8.125778
38	Muc4	8.09526
39	Scgb1c1	8.08462
40	Reg3g	8.054946
41	AU040972	7.880659
42	Ces1a	7.852244
43	Obp1a	7.832625
44	Fmo6	7.809536
45	Cxcl17	7.76269
46	Tmem211	7.752266
47	Dmbt1	7.731351
48	Adam28	7.723887
49	Amelx	7.689168
50	Bpifb3	7.673728

Development • Supplementary information

Table S4. Reagents and resources

REAGENT or RESOURCE	SOURCE	IDENTIFIER
Antibodies		
Rabbit Recombinant Monoclonal anti-SOX9	Abcam	Cat# ab185966, RRID:AB_2728660
Rabbit Polyclonal anti-Osteocalcin	Proteintech	Cat# 23418-1-AP, RRID:AB_2879275
Rabbit Polyclonal anti-Collagen X	Abcam	Cat# ab58632, RRID:AB_879742
Rabbit Polyclonal anti-Collagen II	Thermo Fisher Scientific	Cat# PA1-26206, RRID:AB_779883
Rabbit Monoclonal anti-Cortactin	Abcam	Cat# ab81208, RRID:AB_1640383
Rabbit Polyclonal anti-SH3PXD2	Cosmo Bio	Cat# PRX-MKA4068, RRID:AB_3661885
Mouse Monoclonal anti-ribosomal RNA (Y10b)	Abcam	Cat# ab171119, RRID:AB_2920657
Rabbit Recombinant Monoclonal anti-SOX10	Thermo Fisher Scientific	Cat# 703439, RRID:AB_2809236
Rabbit Monoclonal anti-PCNA	Abcam	Cat# ab92552, RRID:AB_10561973
Goat anti-Rabbit IgG H&L (Alexa Fluor® 488)	Abcam	Cat# ab150077, RRID:AB_2630356
Goat anti-Rabbit IgG H&L (Alexa Fluor™ 647)	Thermo Fisher Scientific	Cat# A-21244, RRID:AB_2535812
Goat Anti-Mouse IgG H&L (Alexa Fluor® 488)	Abcam	Cat# ab150113, RRID:AB_2576208
Chemicals, peptides, and recombinant proteins		
Alexa Fluor™ 594 Phalloidin	Thermo Fisher Scientific	Cat# A12381
Tissue-Tek® O.C.T. Compound	Sakura Finetek Inc.	Cat# 4583
ACK Lysing Buffer	GIBCO	Cat# A1049201
Recombinant Human TGFbeta	Bio Techne	Cat# 7754-BH/CF
TRIzol™ Reagent	Thermo Fisher Scientific	Cat# 15596026
EGTA	EMD Millipore Corp	Cat# 324626
4-Morpholineethanesulfonic acid	Sigma Aldrich	Cat# M3671
Fibronectin bovine plasma	Sigma Aldrich	Cat# F1141

Development • Supplementary information

EmbryoMax® Acidic Tyrode′ s Solution	Sigma Aldrich	Cat# MR-004
Dulbecco′ s Phosphate Buffered Saline	Sigma Aldrich	Cat# D8537
Critical commercial assays		
Click-iT™ EdU Cell Proliferation Kit for Imaging, Alexa Fluor™ 488 dye	Thermo Fisher Scientific	Cat# C10337
In Situ Cell Death Detection Kit, Fluorescein	Roche	Cat# 11684795910
Agilent Small RNA kit	Agilent	Cat# 5067-1548
Experimental models: Cell lines		
Osteoblasts	(Li et al., 2015)	
Dura mater	Li et al (Li et al., 2007)	
Neural crest cells	Gonzalez-Malagon et al (Gonzalez Malagon et al., 2019)[27]<br><br>Moore et al (Moore and Trainor, 2022) [52]	
Experimental models: Organisms/strains		
Mouse *Sh3Pxd2b*^{nee}: B10.Cg-*H2*^{h4} *Sh3pxd2b*^{nee}/GrsrJ	Jackson Laboratory	RRID:IMSR_JAX:006446
Mouse *Wnt1-Cre2*: B6.Cg-*E2f1*$^{Tg(Wnt1-cre)2Sor}$/J	Jackson Laboratory	RRID:IMSR_JAX:022501
Mouse *mT/mG*: B6.129(Cg)-Gt(ROSA)26Sortm4(ACTB-tdTomato,-EGFP)Luo/J	Jackson Laboratory	RRID:IMSR_JAX:007676
Software and algorithms		
GraphPad Prim Version 10.2.2.	GraphPad Software	www.graphpad.com
LAS X	Leica Microsystems	https://www.leica-microsystems.com/products/microscope-software/p/leica-las-x-ls/downloads/
Adobe Photoshop	Adobe Inc.	https://creativecloud.adobe.com/apps/download/photoshop
Adobe Illustrator	Adobe Inc.	https://creativecloud.adobe.com/apps/download/illustrator
ImageJ	Fiji	https://imagej.net/software/fiji/downloads

Development • Supplementary information

NRecon Version 1.7.1.6.	Bruker	N/A
CT Vox Version 3.3.0 r1403 (64-bit)	Bruker	N/A
BioRender	BioRender	www.app.biorender.com
EnrichR	Chen et al, Kuleshov et al, Xie et al (Chen et al., 2013, Kuleshov et al., 2016, Xie et al., 2021)	https://maayanlab.cloud/Enrichr/
Gephi	Bastian et al (Bastian et al., 2009)	http://gephi.org
ToppCluster	Kaimal et al (Kaimal et al., 2010)	http://toppcluster.cchmc.org/
Other		
Leica Inverted Microscope DMI 4000B	Leica Microsystems	N/A
Leica M205FA Fluorescence stereo microscope	Leica Microsystems	N/A
Zeiss LSM880	Carl Zeiss AG	RRID:SCR_017787
Leica SP8	Leica Microsystems	RRID:SCR_017787
NanoDrop™ One/OneC Microvolume UV-Vis Spectrophotometer	Thermo Fisher Scientific	N/A
SkyScan 1276 CMOS Micro CT	Bruker	N/A

Development • Supplementary information

References

BASTIAN, M., HEYMANN, S. & JACOMY, M. 2009. Gephi: An Open Source Software for Exploring and Manipulating Networks. *Proceedings of the International AAAI Conference on Web and Social Media,* 3**,** 361-362.

CHEN, E. Y., TAN, C. M., KOU, Y., DUAN, Q., WANG, Z., MEIRELLES, G. V., CLARK, N. R. & MA'AYAN, A. 2013. Enrichr: interactive and collaborative HTML5 gene list enrichment analysis tool. *BMC Bioinformatics,* 14**,** 128.

GONZALEZ MALAGON, S. G., DOBSON, L., MUNOZ, A. M. L., DAWSON, M., BARRELL, W., MARANGOS, P., KRAUSE, M. & LIU, K. J. 2019. Dissection, Culture and Analysis of Primary Cranial Neural Crest Cells from Mouse for the Study of Neural Crest Cell Delamination and Migration. *J Vis Exp*.

KAIMAL, V., BARDES, E. E., TABAR, S. C., JEGGA, A. G. & ARONOW, B. J. 2010. ToppCluster: a multiple gene list feature analyzer for comparative enrichment clustering and network-based dissection of biological systems. *Nucleic Acids Res,* 38**,** W96-102.

KULESHOV, M. V., JONES, M. R., ROUILLARD, A. D., FERNANDEZ, N. F., DUAN, Q., WANG, Z., KOPLEV, S., JENKINS, S. L., JAGODNIK, K. M., LACHMANN, A., MCDERMOTT, M. G., MONTEIRO, C. D., GUNDERSEN, G. W. & MA'AYAN, A. 2016. Enrichr: a comprehensive gene set enrichment analysis web server 2016 update. *Nucleic Acids Res,* 44**,** W90-7.

LI, S., QUARTO, N. & LONGAKER, M. T. 2007. Dura mater-derived FGF-2 mediates mitogenic signaling in calvarial osteoblasts. *Am J Physiol Cell Physiol,* 293**,** C1834-42.

LI, S., QUARTO, N., SENARATH-YAPA, K., GREY, N., BAI, X. & LONGAKER, M. T. 2015. Enhanced Activation of Canonical Wnt Signaling Confers Mesoderm-Derived Parietal Bone with Similar Osteogenic and Skeletal Healing Capacity to Neural Crest-Derived Frontal Bone. *PLoS One,* 10**,** e0138059.

MOORE, E. L. & TRAINOR, P. A. 2022. Live Imaging of the Dynamics of Mammalian Neural Crest Cell Migration. *Methods Mol Biol,* 2403**,** 263-276.

XIE, Z., BAILEY, A., KULESHOV, M. V., CLARKE, D. J. B., EVANGELISTA, J. E., JENKINS, S. L., LACHMANN, A., WOJCIECHOWICZ, M. L., KROPIWNICKI, E., JAGODNIK, K. M., JEON, M. & MA'AYAN, A. 2021. Gene Set Knowledge Discovery with Enrichr. *Curr Protoc,* 1**,** e90.

Development • Supplementary information
